# Genetic Influence in Exercise Performance

**DOI:** 10.3390/genes12050651

**Published:** 2021-04-27

**Authors:** Juan Del Coso, Alejandro Lucia

**Affiliations:** 1 Centre for Sport Studies, Rey Juan Carlos University, 28943 Fuenlabrada, Spain; juan.delcoso@urjc.es; 2 Faculty of Sport Sciences, Universidad Europea de Madrid, 28670 Villaviciosa de Odón, Spain; 3 Research Institute imas12, Hospital 12 de Octubre, 28041 Madrid, Spain; 4 Centro de Investigación Biomédica en Red de Fragilidad y Envejecimiento Saludable, 28029 Madrid, Spain

Numerous studies in the last two decades have analyzed the association between genetic variants and athletic performance, or other related traits (e.g., responses and adaptations to different exercise modalities or risk of sports injuries). However, the practical applicability of most findings in the field is still limited, with several caveats to consider. Notably, a deeper understanding is needed of potential gene–gene and gene–environment interactions. Future studies should also assess larger samples than in the past, as well as replication cohorts. This Special Issue, entitled *Genetic Influence in Exercise Performance*, includes five original investigations and three reviews that, we think, contribute to building evidence for the potential influence—or actual lack of effect—of some genetic factors on athletic performance.

Yvert and colleagues [1] investigate the individual and combined influence of polymorphisms in the peroxisome proliferator-activated receptor gamma coactivator 1 alpha (*PPARGC1A*) and nuclear respiratory factor 1 (*NRF1)* genes (rs8192678 and rs6949152, respectively) on the muscle fiber composition of healthy individuals. PPARGC1A (commonly known as PGC1α) is a central inducer of mitochondrial biogenesis and function [2]. Previous studies have reported a higher frequency of the rs8192678 G/G genotype in endurance athletes than in their nonathletic controls, suggesting that this genotype might be associated with aerobic performance [3,4,5,6]. In turn, NFR1 is a target of PGC1α whose association with endurance performance has been less studied [7]. In the investigation by Yvert et al. [1], these two polymorphisms were not associated with muscle fiber type distribution—as assessed by the proportion of myosin heavy-chain isoforms—in the *vastus lateralis* of healthy men. Yet, in women, rs8192678 A/A and rs6949152 A/A genotypes showed a link—individually or in combination—with a higher and lower of proportion of I and IIx isoforms, respectively. This investigation suggests that the potential influence of both *PPARGC1A* and *NRF1* genes on endurance performance might be mediated by an effect on muscle fiber differentiation, at least in women. 

Muñoz and colleagues [8] study the influence of the cytochrome P450 family 1 subfamily A member 2 (*CYP1A2*) rs762551 and adenosine A2A receptor (*ADORA2A*) rs5751876 polymorphisms on the ergogenic effect of acute caffeine intake in professional handball players. The CYP1A2 enzyme is responsible for ~95% of caffeine metabolism, and individuals with the rs762551 A/A genotype are categorized as “fast metabolizers” because they usually show a higher rate of caffeine metabolization into paraxanthine than their referents with the C-allele [9]. There is also rationale to support a role for the *ADORA2A* gene because a major mechanism explaining the potential ergogenic effect of caffeine at the brain level is a blockade of the receptors for an inhibitory neurotransmitter, adenosine, thereby increasing arousal [10]. In this context, *ADORA2A* T/T are potentially “high-responders” to caffeine compared to those with the C-allele [11]. Although both *CYPA1A2* A/A [12] and *ADORA2A* T/T [13] individuals might show a higher ergogenic effect of caffeine ingestion than those with the C-allele, the potential influence of these gene variants is rather small [14,15,16,17]. In the Muñoz et al. study [8], the neuromuscular performance of handball players increased with the intake of 3 mg/kg of caffeine, irrespective of *CYPA1A2* and *ADORA2A* genotype. Thus, this investigation contributes to the body of knowledge on the ergogenic effect of caffeine on sports performance in real sport scenarios, and it does not support a role for *CYPA1A2* or *ADORA2A* genetic variants. In this regard, we suggest that, although they might appear less attractive or even less publishable, “negative” findings are as important as positive ones in order to build a balanced perspective of the scientific evidence. If only “positive” findings were to be published, we would only see the tip of the iceberg in the nurture vs. nature debate. 

Lulińska and colleagues [18] study the association of variants in several matrix metalloproteinases (MMP)-encoding genes (*MMP1* rs1799750, *MMP10* (rs486055) and *MMP12* (rs2276109)) and the risk of an important injury in the context of sports—non-contact anterior cruciate ligament (ACL) rupture. These authors address a relevant question. Indeed, the prevention of ACL injuries is essential, given the long time lost for competition, the long-term consequences on articular cartilage, even in surgically treated injuries, and the resulting high economic costs [19]. MMPs play an essential role in the homeostasis of the extracellular matrix (ECM) of connective tissue [20], as these enzymes digest collagen and other structural molecules within ECM [21], with genetic MMP gene variants potentially affecting the capacity of connective tissue to bear mechanical force due to excessive collagen degradation. For instance, it has been previously reported that the rs2276109 A/A genotype might be associated with a higher risk of non-contact ACL rupture [22], with some interactions reported for the genes encoding the different types of collagen in the context of ACL rupture risk [23,24]. However, Lulińska et al. report no association of the aforementioned *MPP* variants with non-contact ACL rupture when comparing genotype distributions in individuals with primary non-contact ACL rupture vs. their referents without any history of this condition. Thus, their findings do not support the use of genetic screening among athletes to prevent ACL rupture (i.e., by identifying potential “high-risk” athletes), which is in line with recent research [25]. 

Leońska-Duniec and colleagues [26] investigate the potential influence of the c.34C/T polymorphism (rs17602729) in the gene that codifies the skeletal-muscle isoform of adenosine monophosphate deaminase (*AMPD1*) on the effects of a 12-week aerobic training program in obesity related parameters among healthy women. This enzyme plays an important regulatory role in muscle metabolism by catalyzing the deamination of adenosine monophosphate (AMP) to inosine monophosphate (IMP) and shifting the reaction towards ATP resynthesis in muscles. In fact, the aforementioned polymorphism has been previously linked with endurance performance, with the C/C genotype associated with a higher likelihood of being an elite endurance athlete vs. carriage of the mutant T-allele [4,27,28]. Additionally, T/T and C/T individuals might show a significantly lower relative increase in cardiorespiratory fitness (peak oxygen uptake, VO_2peak_) after three months of training compared to their C/C counterparts [29], which is in overall agreement with a previous report from the classic HERITAGE Family study reporting a lower trainability of aerobic fitness in T/T individuals compared to the two other genotypes [30]. Interestingly, although the underlying mechanisms remain to be elucidated, there is preliminary evidence for a certain role of the c.34C/T variation in cardiometabolic traits [31]. In the Leońska-Duniec et al. study [26], a 12-week training program proved effective in producing a significant loss of body mass and relative fat mass while increasing fat-free mass and high-density lipoprotein cholesterol, as well as improving glucose control. However, these benefits were of similar magnitude irrespective of c.34C/T genotype. These results suggest that the T allele in the c.34C/T polymorphism does not influence exercise training responses with regard to body composition and metabolic parameters, at least in healthy women. 

Determining the potential influence of genetic factors on VO_2peak_ is of medical relevance given the strong, independent, and predictive value of this variable with regard to cardiovascular risk and mortality. A seminal study by Claude Bouchard and colleagues found comparable VO_2peak_ values in brothers of the same sibship, and the similarities in VO_2peak_ were even greater in dizygotic and monozygotic twins [32], with the authors suggesting that the genetic effect on VO_2peak_ reached ~40%. In this issue of the journal, Gaowa and colleagues [33] study the influence of several candidate genetic variants in VO_2peak_ among ~1000 untrained Han Chinese people, with a similar representation of both sexes. When setting a stringent threshold p-value of 0.0004, only one single nucleotide polymorphism (rs4295), located in the gene encoding angiotensin-converting enzyme, was associated with VO_2peak_. Furthermore, from a list of 125 candidate variants showing previous evidence for an association with exercise or cardiovascular traits, only three (rs4295 = 1.1%, angiotensin II receptor type 1 rs275652 = 0.6%, and myostatin rs7570532 = 0.5%) accounted together for just 2.2% (*p* = 0.0007) of the interindividual variance in VO_2peak_. The authors conclude that a strong independent health indicator, such as cardiorespiratory fitness, might be influenced by several polymorphic variations in candidate genes (at least in the pre-trained state), but these polymorphisms represent a minor portion of the variance, suggesting a more important role for environmental factors. An important novelty of this study is the ethnic origin of the population, as the bulk of the evidence on genetics and exercise-related traits comes from research conducted in Caucasian people, and these findings might not be extrapolated to other ethnicities.

There are three high-quality reviews in this Special Issue. Kitazawa and coworkers [34] discuss the importance of central nervous system (CNS)-related genes and athletic performance. These authors neatly review the role of some CNS areas and functions related to a predisposition to inherent exercise capacity, such as the pain analgesia system, the dopaminergic and serotonergic systems, neuronal circuits related to neuroplasticity, and thermoregulation-related factors. This review also focuses on CNS-related variants that might be involved in exercise capacity, such as those related to the serotonergic system, as well as the GABAergic, glutamatergic, dopaminergic, or noradrenergic systems (e.g., the fifth Ewing variant (*FEV*) in the serotonergic system, D2 receptor (*DRD2*) and catechol-O-methyltransferase (*COMT*) in the dopaminergic system, and potentially transcranial direct current stimulation tDCS-responsive genes in the GABAergic system). Future research ideas are also discussed. On the other hand, Hall et al., [35] propose a novel contextual framework in sports sciences—the use of chromosome conformation signature (CCS). CCS refers to the organization of the human genome within three-dimensional space, which has recently emerged as a dynamic epigenetic regulator of gene expression. In this interesting review, the authors also show examples of how CCS data may help to explain interindividual differences in response to exercise or nutritional interventions, and could also be helpful for potential doping detection. Finally, Pickering et al., [36] discuss why it is still too early to use genetic information for talent detection in sports based on scientific evidence; this is despite the dozens of commercially available genetic tests that are purported to predict sports performance. Other tools (e.g., “genotype scores”) are also proposed to help identify those individuals who might be inherently predisposed to succeed in sport.

Although the field of sports genetics is fascinating, the task of identifying “the champions’ genes” is a very difficult one. Much more research is needed before genetic screening becomes a useful tool in the context of personalized exercise training for disease treatment or prevention.
